# MAPRE2 is associated with macrophage-enriched innate immune dysregulation and malignant phenotypes in hepatocellular carcinoma

**DOI:** 10.3389/fimmu.2026.1849407

**Published:** 2026-05-14

**Authors:** Xiuqin An, Yanfang Gao, Shangyumeng Zhao, Wei He, Xinda Yang, Yujie Wu, Jihao Li, Runxi Yi, Yue Han, Mingxuan Li, Qiusheng Li, Ting Yang

**Affiliations:** 1 Department of Gastroenterology, First Hospital of Shanxi Medical University, Taiyuan, Shanxi, China; 2 Department of Gastroenterology, Shanxi Provincial People’s Hospital, Taiyuan, Shanxi, China; 3 Department of Preventive Medicine, College of Public Health, Hebei Medical University, Shijiazhuang, China; 4 Department of Hepatobiliary Surgery, The Second Hospital of Hebei Medical University, Shijiazhuang, China

**Keywords:** complement, hepatocellular carcinoma, innate immunity, macrophages, MAPRE2, Mendelian randomization, single-cell RNA sequencing, tumor microenvironment

## Abstract

**Background:**

Hepatocellular carcinoma (HCC) remains difficult to treat, and molecular determinants that link malignant behavior to the innate immune microenvironment are still needed.

**Methods:**

We integrated two-sample Mendelian randomization using cis-eQTL and cis-pQTL instruments with tumor transcriptomics, immune deconvolution, DNA methylation analyses, single-cell RNA sequencing, and loss-of-function assays to prioritize and characterize MAPRE2 in HCC.

**Results:**

Multi-omics Mendelian randomization identified MAPRE2 as a concordant risk-increasing candidate across transcript and protein layers. MAPRE2 was overexpressed in tumors and associated with worse overall survival. MAPRE2-high tumors were enriched for complement activation, TNFA signaling via NF-kB, interferon gamma response, extracellular matrix remodeling, and epithelial-mesenchymal transition, and showed stronger macrophage-, neutrophil-, and dendritic-cell-related signatures together with lower immunophenoscores, consistent with an inflamed but immunosuppressed innate immune microenvironment. Single-cell analysis showed multicompartment expression of MAPRE2, and virtual knockout implicated metabolic, adhesion, and extracellular programs. DNA methylation was associated with MAPRE2 expression and outcome, whereas recurrent high-level somatic amplification was rare. In HepG2 and MHCC97 cells, MAPRE2 knockdown suppressed proliferation, invasion, and wound closure.

**Conclusion:**

MAPRE2 marks an aggressive HCC-associated molecular state coupled to macrophage-enriched innate immune dysregulation and may represent a candidate biomarker and therapeutic target that warrants rescue, *in vivo*, transplant-cohort, and external prognostic validation.

## 1 Introduction

Hepatocellular carcinoma (HCC) is the predominant histologic subtype of primary liver cancer and remains a leading cause of cancer-related death worldwide (1). Curative treatments such as resection, ablation, or transplantation benefit only a minority of patients, recurrence after local therapy is common, and patients with advanced disease still face limited durable benefit despite recent progress in systemic therapy (1). This therapeutic bottleneck highlights the need for new biomarkers and molecular targets that capture the biology of aggressive HCC more faithfully. Because the liver is an immunologically specialized organ enriched in macrophages, neutrophils, dendritic cells, and complement pathways, identifying tumor drivers linked to innate immune dysregulation may be particularly informative for HCC progression and treatment resistance (2).

Mendelian randomization (MR) provides a complementary strategy for prioritizing such candidates by using inherited regulatory variants as instrumental variables (3, 4). When cis-eQTLs or cis-pQTLs are used as exposure instruments, MR can estimate whether lifelong genetically predicted differences in transcript or protein abundance influence disease susceptibility (5, 6). In cancer research, this approach is attractive because it anchors inference to germline-regulated biology rather than to the unstable expression patterns observed in established tumors. In principle, convergence across transcript-level and protein-level QTL datasets can highlight genes that are not only differentially expressed, but also genetically supported and therefore more plausible candidates for downstream mechanistic and therapeutic investigation.

MAPRE2 (also known as EB2) encodes a microtubule plus-end tracking protein involved in cytoskeletal organization, focal adhesion dynamics, and cell motility (7). Previous work suggested that EB2 promotes HCC proliferation and metastasis through MAPK/ERK signaling (8), but its relationship to the innate immune microenvironment of HCC remains poorly understood. Here, we integrated multi-omics Mendelian randomization, bulk transcriptomics, immune deconvolution, single-cell RNA sequencing, methylation and protein analyses, and loss-of-function experiments to determine whether MAPRE2 is linked to innate immune dysregulation and malignant progression in HCC.

## 2 Materials and methods

### 2.1 Public datasets and overall study design

RNA sequencing count data and clinicopathologic information for TCGA-LIHC were obtained from the Genomic Data Commons and included 374 tumor tissues and 50 solid normal liver tissues (2). Transcript-level cis-eQTL summary statistics were obtained from the eQTLGen consortium (5), and plasma cis-pQTL summary statistics were obtained from the deCODE proteogenomic resource (6). HCC genome-wide association summary statistics were downloaded from the GWAS Catalog under accession GCST90043858 (9). Single-cell RNA sequencing data were obtained from GEO accession GSE149614 (10). Additional somatic, methylation, pharmacogenomic, and proteomic interrogation used cBioPortal, GSCA, and UALCAN/CPTAC. The overall workflow consisted of candidate-gene prioritization by multi-omics MR, variant-level visualization of MAPRE2 instruments, characterization of somatic and regulatory context, bulk and single-cell tumor analyses, and *in vitro* validation.

### 2.2 Differential expression analysis and candidate gene selection

Bulk RNA sequencing count matrices from TCGA-LIHC were analyzed in R with DESeq2 (11). Genes significantly different between tumor and normal liver tissues after multiple-testing correction were considered differentially expressed genes and were intersected with genes represented in the cis-eQTL and cis-pQTL resources. The resulting overlap constituted the instrumentable candidate gene set used for Mendelian randomization.

### 2.3 Two-sample Mendelian randomization and single-variant analyses

Instrumental variables for the targeted genes were constructed based on independent, genome-wide significant cis-QTLs linked to either transcript or protein levels. To ensure genetic independence, linkage disequilibrium clumping was executed (r^2^ < 0.1, distance = 10,000 kb), alongside the strict elimination of palindromic SNPs with intermediate allele frequencies during the data harmonization stage. Instrument strength was evaluated by the F statistic, and weak instruments were excluded. The primary causal estimate was obtained using the inverse-variance weighted method (3). Sensitivity analyses included MR-Egger regression, weighted median, weighted mode, and simple mode estimators (4). Horizontal pleiotropy and heterogeneity were additionally evaluated using standard MR diagnostics, and Steiger directionality testing was used to confirm the exposure-to-outcome direction (12, 13). Genes were retained as high-confidence candidates when the eQTL-based and pQTL-based inverse-variance weighted estimates were both nominally significant and pointed in the same direction.

For MAPRE2, the retained cis-eQTL and cis-pQTL instruments were further visualized by single-variant MR plots to display instrument-level effect estimates and confidence intervals. Crucially, all genetic instrumental variables utilized in this study were strictly annotated according to the Human Genome Variation Society (HGVS) nomenclature guidelines based on the GRCh38 assembly (reference sequence NC_000018.10 for MAPRE2). Detailed variant-level outputs, including complete HGVS genomic coordinates and effect sizes, are compiled in **Supplementary Table 1**.

### 2.4 Somatic alteration, methylation, protein, and pharmacogenomic analyses

MAPRE2 somatic alterations in TCGA-LIHC were explored in cBioPortal (14). The default OncoPrint display was used to summarize rare high-level events, and a representative screenshot is shown as **Supplementary Figure 1**. GSCA (15) was used to summarize MAPRE2 copy-number frequencies, evaluate CNV-expression and SNV-expression relationships, assess differential methylation between tumor and normal samples, test methylation-expression and methylation-survival associations, and perform exploratory pharmacogenomic analyses using CTRP and GDSC. MAPRE2 protein abundance in normal liver and primary HCC was assessed using CPTAC-based data available through UALCAN (16).

### 2.5 Tumor transcriptome, survival, and immune analyses

For downstream characterization, TCGA-LIHC tumors were dichotomized into MAPRE2-high and MAPRE2-low groups. Median MAPRE2 expression was used for transcriptomic stratification and immune analyses, whereas the cutoff shown in the survival analysis was chosen by maximization of the log-rank statistic. Differential expression between MAPRE2-high and MAPRE2-low tumors was assessed with limma (17). Gene Ontology enrichment was performed with clusterProfiler (18), and hallmark gene set enrichment analysis was performed against the MSigDB hallmark collection (19).

Immune cell composition was inferred with two complementary approaches. CIBERSORT with the LM22 leukocyte reference matrix was used to estimate immune cell fractions (20). Single-sample gene set enrichment analysis was performed with GSVA/ssGSEA to score published immune cell signatures (21). Immunophenoscores were obtained from The Cancer Immunome Atlas (22). For IPS analyses, tumors were stratified into four immune subgroups according to CTLA4 and PD1 status, and MAPRE2-high versus MAPRE2-low tumors were compared within each subgroup. Overall survival was evaluated by Kaplan-Meier analysis with log-rank testing, and hazard ratios with 95% confidence intervals were derived from Cox regression. Because the survival analysis was exploratory and not designed as a pre-specified prognostic model, MAPRE2 was interpreted as an HCC-associated candidate marker rather than a validated independent prognostic predictor.

### 2.6 Single-cell RNA sequencing analysis and virtual knockout

The GSE149614 single-cell RNA sequencing dataset was processed in Seurat (23). Only cells derived from primary tumor samples were retained. Cells with low feature counts, very small library size, or excessive mitochondrial transcripts were removed using standard quality-control criteria, and genes detected in very few cells were filtered out. Data were normalized with the LogNormalize workflow, 2,000 highly variable genes were identified, and dimensionality reduction was performed by principal component analysis followed by UMAP visualization. Cell clusters were annotated according to canonical markers into T/NK, myeloid, endothelial, hepatocyte, fibroblast, and B-cell populations.

To explore the putative regulatory role of MAPRE2, a virtual knockout analysis was performed with scTenifoldKnk (24). A matrix containing the top 1,000 highly variable genes across tumor-derived microenvironment cells was used to construct the baseline single-cell gene regulatory network. MAPRE2 was then virtually deleted from the network, and the pseudo-knockout network was compared with the wild-type network by manifold alignment. Genes with significant knockout shifts were considered MAPRE2-responsive, and functional enrichment analysis was performed on these genes.

### 2.7 Cell culture and siRNA transfection

The following human hepatocellular carcinoma cell lines were used in this study: Hep3B (RRID: CVCL_0326), Huh7 (RRID: CVCL_0336), MHCC97 (RRID: CVCL_4972), and HepG2 (RRID: CVCL_0027). All cell lines were officially obtained from the Cell Bank of the Chinese Academy of Sciences (Shanghai, China). Cells were maintained in DMEM containing 10% FBS alongside a 1% antibiotic mixture (penicillin/streptomycin) under standard incubation conditions at 37 °C with a 5% CO_2_ atmosphere. All cell lines were routinely monitored and tested for mycoplasma contamination using a commercially available PCR-based mycoplasma detection kit prior to the experiments and confirmed to be free of contamination. Two independent small interfering RNAs targeting MAPRE2 (si-1 and si-2) and a non-targeting negative control were transfected into HepG2 and MHCC97 cells using a lipid-based transfection reagent according to the manufacturer’s instructions.

### 2.8 RT-qPCR and western blotting

RNA isolation was performed applying the TRIzol method, followed by cDNA synthesis. Transcript levels were subsequently evaluated via quantitative PCR employing a SYBR Green-based fluorescent system. MAPRE2 expression was normalized to GAPDH and calculated with the 2^-ΔΔCt^ method. The primer sequences used for MAPRE2 were forward 5’-GTCAGACGCAGCACCTACTT-3’ and reverse 5’-TAGGCCGCTCCTGAACAAAG-3’.

For immunoblotting, cells were lysed in RIPA buffer supplemented with protease inhibitors. Protein concentrations were measured by BCA assay. Equivalent protein quantities underwent SDS-PAGE separation and were electro-transferred onto PVDF membranes. Following a blocking step in 5% defatted milk, the blots were probed with anti-MAPRE2 or anti-ACTIN primary antibodies, washed, and then treated with HRP-linked secondary antibodies. Bands were visualized by enhanced chemiluminescence and quantified by densitometry.

### 2.9 Cell proliferation, invasion, and migration assays

To monitor cell viability, the siRNA-treated HCC populations were distributed into 96-well microplates. Cellular growth was evaluated daily over a consecutive four-day period by introducing the Cell Counting Kit-8 (CCK-8) solution to the cultures. Following a designated incubation phase, the optical density for each well was determined at a wavelength of 450 nm utilizing a microplate reader.

The invasive potential of the cells was assessed using Transwell inserts pre-coated with a layer of Matrigel. Specifically, cell suspensions prepared in basal medium (lacking serum) were loaded into the apical compartments, whereas the basolateral chambers were filled with complete medium (containing 10% FBS) to serve as a nutritional chemoattractant. After a 24-hour incubation period, cells that had successfully traversed to the reverse side of the porous membrane were immobilized, visualized via crystal violet staining, and subsequently enumerated under a light microscope.

To examine cellular migration, we conducted *in vitro* scratch assays. Transfected cells were grown in six-well culture plates until they reached approximately 90% to 100% confluence. Artificial wound gaps were generated across the uniform cell monolayers using a sterile plastic pipette tip. After gently rinsing away any detached cellular debris with PBS, the adherent cells were maintained in a low-serum environment. Microscopic photographs of the specific wounded zones were captured both at baseline (0 h) and post-incubation (24 h). The extent of cell migration was conclusively evaluated by calculating the unclosed gap area at the 24-hour mark.

### 2.10 Statistical analysis

Public dataset analyses were performed in R, and cell-based experiments were analyzed in GraphPad Prism. *In vitro* results are displayed as the mean ± standard error of the mean (SEM) derived from triplicate biological replicates. Differences between two independent cohorts were evaluated utilizing either the Student’s t-test or the Wilcoxon rank-sum test, depending on data distribution. For assessments involving multiple groups, a one-way ANOVA with Tukey’s *post-hoc* evaluation was applied, whereas a two-way ANOVA was utilized for evaluating proliferation trajectories. Correlation metrics were calculated using Spearman’s approach. Statistical significance was defined at a two-tailed p-value below 0.05.

## 3 Results

### 3.1 Multi-omics Mendelian randomization prioritizes MAPRE2 as a genetically supported HCC susceptibility candidate

**Figure 1:**
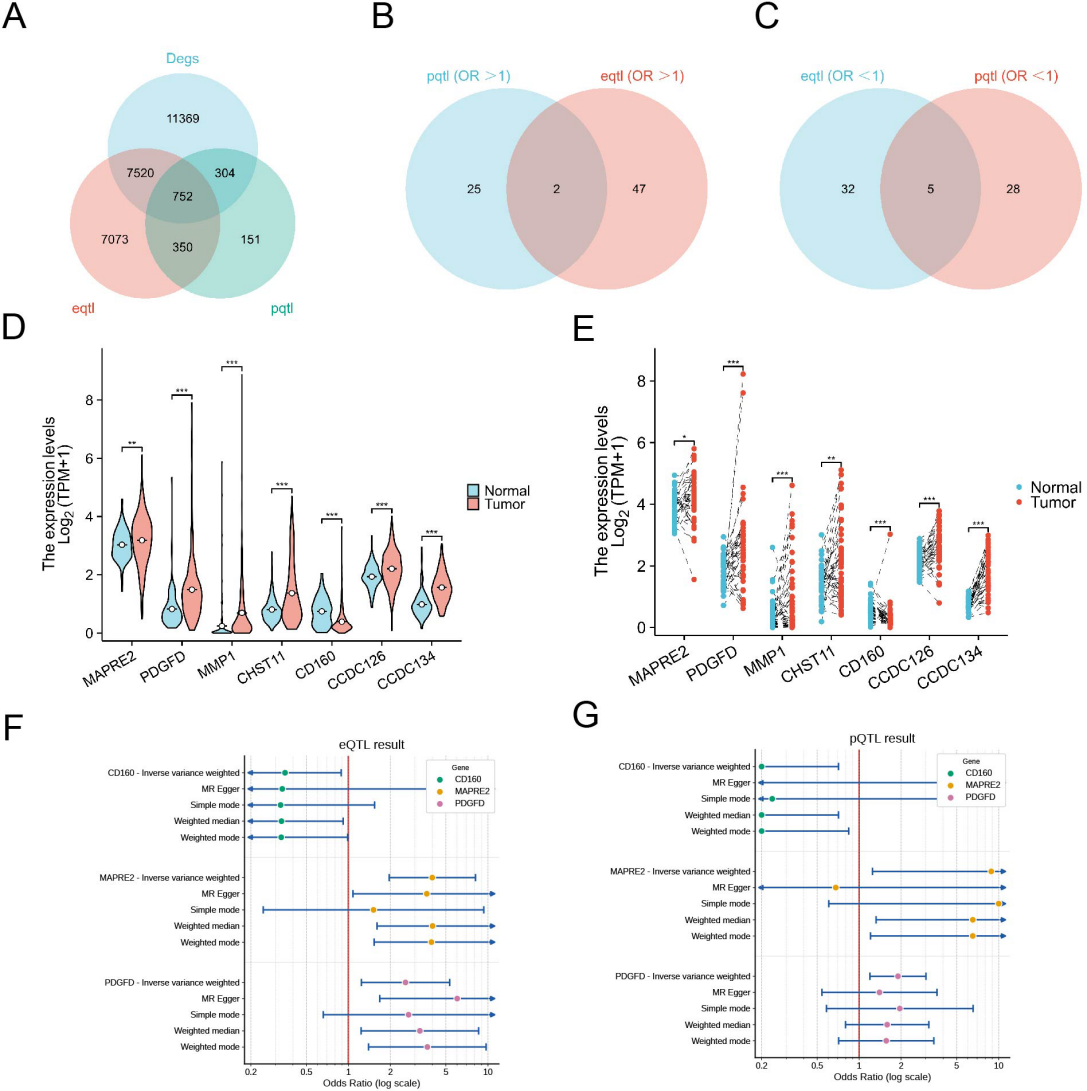
Multi-omics Mendelian randomization prioritizes candidate causal genes for hepatocellular carcinoma. **(A)** Venn diagram showing overlap among differentially expressed genes in TCGA-LIHC and genes covered by the cis-eQTL and cis-pQTL resources. The three-way overlap contained 752 candidate genes. **(B)** Overlap of genes with concordant risk estimates, defined as odds ratios greater than 1 in both pQTL-based and eQTL-based Mendelian randomization analyses. **(C)** Overlap of genes with concordant protective estimates, defined as odds ratios less than 1 in both eQTL-based and pQTL-based Mendelian randomization analyses. **(D)** Unpaired tumor versus normal expression comparison for the seven concordant genes in TCGA-LIHC. **(E)** Paired tumor-normal expression comparison for the same seven genes. **(F)** Forest plot of representative eQTL-based Mendelian randomization estimates for CD160, MAPRE2, and PDGFD across inverse-variance weighted, MR-Egger, simple mode, weighted median, and weighted mode methods. **(G)** Corresponding pQTL-based Mendelian randomization estimates for CD160, MAPRE2, and PDGFD. In **(F, G)** horizontal lines indicate 95% confidence intervals and the red vertical line denotes an odds ratio of 1. *P < 0.05, **P < 0.01, ***P < 0.001.

To identify genes with potential causal relevance to hepatocellular carcinoma, we first intersected differentially expressed genes from TCGA-LIHC with genes represented in large cis-eQTL and cis-pQTL resources. This three-way integration yielded 752 candidate genes supported by tumor expression data, transcript-level genetic regulation, and protein-level genetic regulation (**Figure 1A**).

Two-sample Mendelian randomization was then performed for the 752 genes using cis-eQTLs or cis-pQTLs as exposure instruments and HCC summary statistics as the outcome. A total of 86 genes reached nominal significance in the eQTL-based analysis, whereas 60 genes reached nominal significance in the pQTL-based analysis. When we further required directional concordance across the two molecular layers, two genes, MAPRE2 and PDGFD, showed concordant risk estimates with odds ratios greater than 1, and five genes, MMP1, CHST11, CD160, CCDC126, and CCDC134, showed concordant protective estimates with odds ratios less than 1 (**Figures 1B, C**). Representative forest plots across multiple robust MR methods (including inverse-variance weighted, MR-Egger, and weighted median) confirmed the consistent risk-increasing effects of MAPRE2 and PDGFD, as well as the protective effect of CD160 (**Figures 1F, G**).

Tumor transcriptomic analysis refined this set further. In unpaired TCGA comparisons, MAPRE2, PDGFD, MMP1, CHST11, CCDC126, and CCDC134 were significantly increased in tumors, whereas CD160 was decreased (**Figure 1D**). Paired tumor-normal comparisons showed the same overall expression pattern (**Figure 1E**). Thus, only MAPRE2 and PDGFD showed agreement between risk direction in Mendelian randomization and tumor upregulation, whereas CD160 showed agreement between protective direction and lower tumor expression. Because MAPRE2 combined concordant MR support with reproducible tumor overexpression and adverse clinical associations, it was selected for detailed downstream characterization.

### 3.2 Single-variant Mendelian randomization supports MAPRE2 across multiple cis-regulatory instruments

**Figure 2:**
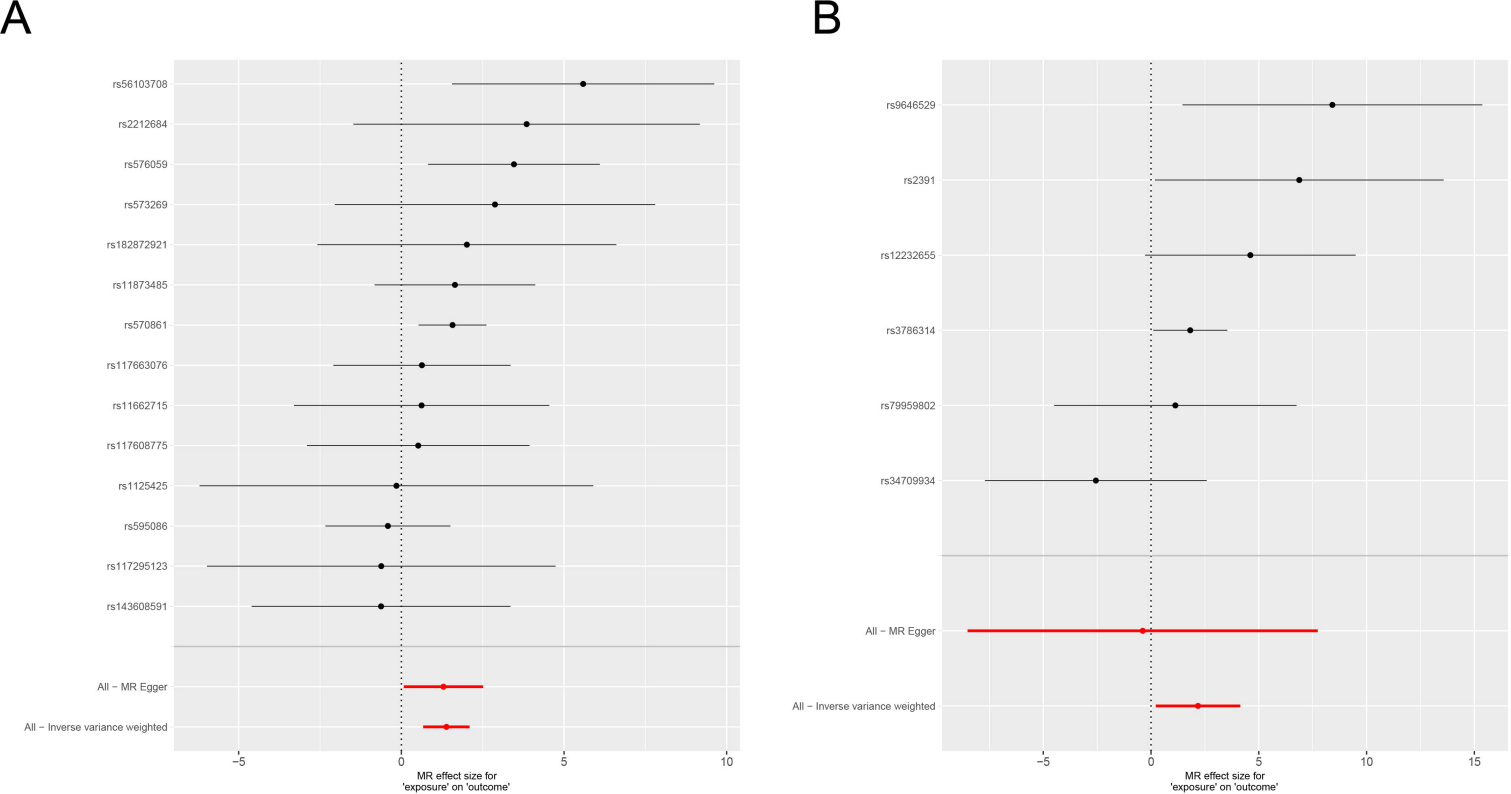
Single-variant Mendelian randomization estimates for MAPRE2 in hepatocellular carcinoma. **(A)** Instrument-level causal estimates for retained MAPRE2 cis-eQTL variants against HCC risk. **(B)** Instrument-level causal estimates for retained MAPRE2 cis-pQTL variants against HCC risk. Black points and horizontal lines indicate single-variant estimates with 95% confidence intervals. Red points and horizontal lines indicate the combined inverse-variance weighted and MR-Egger estimates. The dotted vertical line marks the null effect.

We next visualized instrument-level estimates for MAPRE2 to make the genetic support more explicit at the variant level. In the eQTL-based analysis, 14 retained cis-eQTL instruments were available. Most point estimates were positive, including core risk-conferring variants such as rs56103708, rs2212684, rs576059, and rs573269 (detailed HGVS notations provided in **Supplementary Table 1**), and the combined inverse-variance weighted estimate also remained positive (**Figure 2A**). The MR-Egger estimate was directionally similar but less precise, as expected from the smaller effective information content of this method.

In the pQTL-based analysis, 6 retained cis-pQTL instruments were available. rs9646529, rs2391, and rs12232655 showed positive point estimates, and the combined inverse-variance weighted estimate again remained in the positive direction (**Figure 2B**). Although confidence intervals for individual variants were broad, the overall pattern was directionally concordant across transcript-level and protein-level instruments. These plots therefore strengthen the interpretation that MAPRE2 is not merely a gene-level statistical hit, but a locus supported by multiple regulatory variants. Detailed variant-level outputs are provided in the accompanying supplementary Excel file.

### 3.3 MAPRE2 dysregulation in LIHC is linked to inherited regulatory and epigenetic signals rather than recurrent high-level somatic amplification

A genetically prioritized susceptibility gene is not necessarily a recurrent tumor hotspot. We therefore examined whether somatic genomic changes could explain MAPRE2 dysregulation in LIHC. In cBioPortal, the default OncoPrint display showed that MAPRE2 high-level amplification was rare, occurring in only 1 of 379 profiled samples (<1%; **Supplementary Figure 1**). GSCA-based copy-number summaries likewise suggested that MAPRE2 genomic events were dominated by low-level heterozygous changes rather than by high-level alterations: total amplification 7.57%, total deletion 21.35%, heterozygous amplification 7.30%, heterozygous deletion 21.35%, homozygous amplification 0.27%, and homozygous deletion 0.00%. Importantly, CNV did not significantly correlate with MAPRE2 mRNA expression (Spearman = 0.04, FDR = 0.62), and SNV-expression association was also not significant. These data argue against recurrent somatic SNV/CNV as the primary explanation for MAPRE2 upregulation in LIHC.

**Figure 3:**
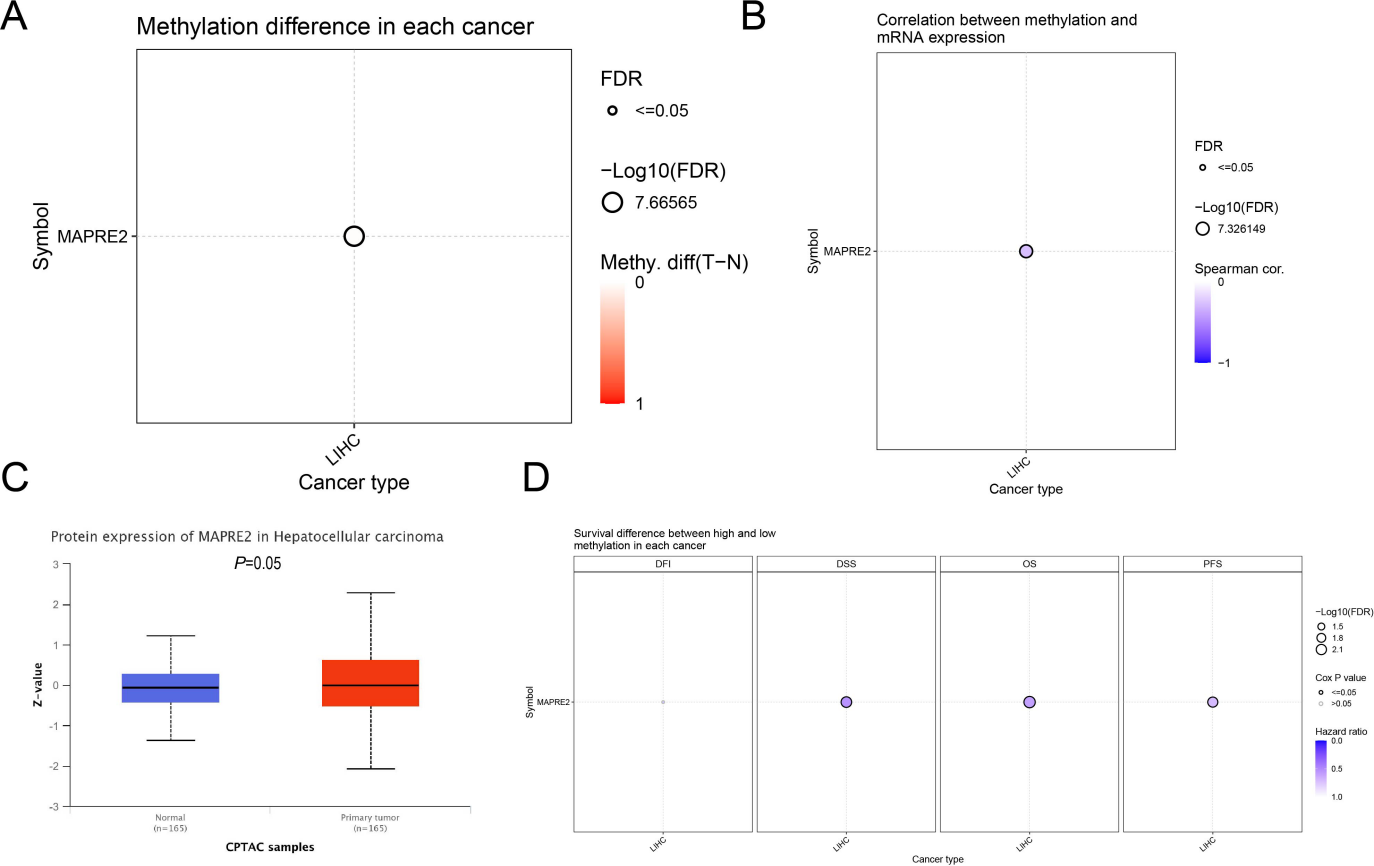
Epigenetic and protein-level evidence for MAPRE2 dysregulation in liver hepatocellular carcinoma. **(A)** Differential methylation analysis for a MAPRE2-associated CpG site between tumor and normal LIHC samples from GSCA. **(B)** Correlation between MAPRE2-associated methylation and MAPRE2 mRNA expression in LIHC. **(C)** CPTAC/UALCAN protein abundance of MAPRE2 in normal liver and primary liver tumor samples. **(D)** Survival differences between high- and low-methylation groups for DFI, disease-free interval; DSS, disease-specific survival; OS, overall survival; PFS, progression-free survival in LIHC.

By contrast, epigenetic and protein-level analyses suggested a more plausible regulatory context. GSCA showed a significant but small tumor-normal methylation difference for a MAPRE2-associated CpG site in LIHC (tumor - normal difference = 0.01, FDR = 2.16e-8; **Figure 3A**). Within LIHC, methylation at cg13550107 was inversely correlated with MAPRE2 mRNA expression (Spearman = -0.28, FDR = 4.72e-8; **Figure 3B**). Lower methylation was also associated with worse disease-specific survival, overall survival, and progression-free survival, with a similar but borderline trend for disease-free interval (**Figure 3D**). At the proteomic level, CPTAC/UALCAN showed borderline higher MAPRE2 protein abundance in primary liver tumors than in normal liver tissue (P = 0.05; **Figure 3C**). Taken together, these findings support a model in which inherited regulatory variants and epigenetic mechanisms may contribute to MAPRE2 dysregulation in LIHC, whereas recurrent high-level somatic amplification or SNV events are unlikely to be the primary explanation.

### 3.4 MAPRE2-high tumors display aggressive transcriptomic programs and innate immune dysregulation

**Figure 4:**
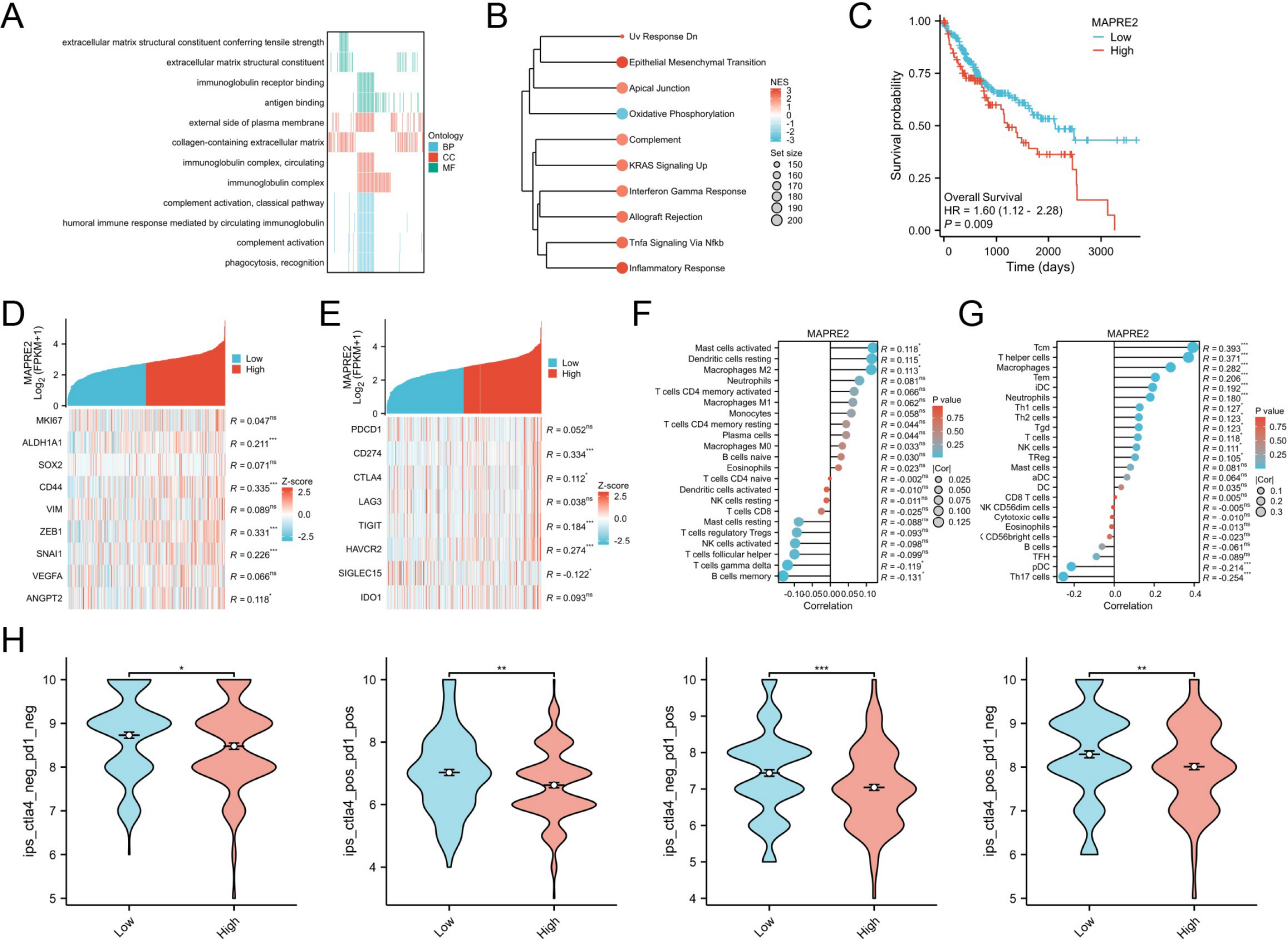
MAPRE2-high tumors display aggressive transcriptomic programs, immune checkpoint expression, and reduced immunogenicity in hepatocellular carcinoma. **(A)** Gene Ontology enrichment analysis of MAPRE2-associated genes. **(B)** Hallmark gene set enrichment analysis comparing MAPRE2-high and MAPRE2-low tumors. **(C)** Kaplan-Meier overall survival analysis according to MAPRE2 expression. **(D)** Heatmap of selected malignant phenotype-related genes across tumors ranked by MAPRE2 expression. **(E)** Heatmap of selected immune checkpoint and immune modulatory genes across tumors ranked by MAPRE2 expression. **(F)** Correlation between MAPRE2 expression and CIBERSORT-estimated immune cell fractions. **(G)** Correlation between MAPRE2 expression and ssGSEA-derived immune signatures. **(H)** Comparison of immunophenoscore values between MAPRE2-high and MAPRE2-low tumors within CTLA4/PD1-defined immune subgroups. *P < 0.05, **P < 0.01, ***P < 0.001; ns, not significant.

We next examined the biological context of MAPRE2 expression in bulk tumor transcriptomes. Gene Ontology analysis of genes associated with MAPRE2 highlighted extracellular matrix structural constituents, collagen-containing extracellular matrix, immunoglobulin complexes, complement activation, and phagocytosis-related terms (**Figure 4A**). Hallmark gene set enrichment analysis showed enrichment of epithelial-mesenchymal transition, apical junction, complement, KRAS signaling up, interferon gamma response, allograft rejection, TNFA signaling via NFκB, inflammatory response, and UV response down in MAPRE2-high tumors, whereas oxidative phosphorylation showed the opposite pattern (**Figure 4B**). These results place MAPRE2 within an aggressive transcriptomic program characterized by matrix remodeling and inflammatory stromal activity.

High MAPRE2 expression was also associated with inferior clinical outcome. Kaplan-Meier analysis showed that patients in the MAPRE2-high group had significantly shorter overall survival than those in the MAPRE2-low group, with a hazard ratio of 1.60 (95% confidence interval 1.12-2.28; P = 0.009) (**Figure 4C**). Because this retrospective survival analysis was exploratory and was not designed as a pre-specified prognostic model, this association was interpreted as supportive evidence for MAPRE2 as an HCC-associated candidate marker rather than as proof of independent predictive value. Consistent with a more invasive tumor state, tumors ranked by MAPRE2 expression showed positive associations for several malignant phenotype-related markers, particularly ALDH1A1, CD44, ZEB1, SNAI1, and ANGPT2 (**Figure 4D**).

Because the enrichment results suggested substantial immune involvement, we next assessed immune modulatory genes and infiltration patterns. Tumors with higher MAPRE2 expression showed stronger expression of several checkpoint or immune-suppressive genes, including CD274, CTLA4, TIGIT, and HAVCR2, while LAG3, IDO1, and SIGLEC15 displayed weaker associations (**Figure 4E**). CIBERSORT showed positive correlations between MAPRE2 and activated mast cells, resting dendritic cells, neutrophils, and notably M2-like macrophage fractions (**Figure 4F**). A second immune-signature analysis likewise supported positive associations with macrophage-related and helper/memory T-cell programs, while Th17 cells and plasmacytoid dendritic cells were negatively associated (**Figure 4G**). Across all four CTLA4/PD1-defined immune subgroups, MAPRE2-high tumors showed lower immunophenoscores than MAPRE2-low tumors (**Figure 4H**). Thus, MAPRE2 expression was associated not simply with inferred immune-cell enrichment, but with an inflamed yet potentially immunosuppressed tumor state.

### 3.5 Single-cell RNA sequencing and virtual knockout analyses suggest multicompartment functions of MAPRE2

**Figure 5:**
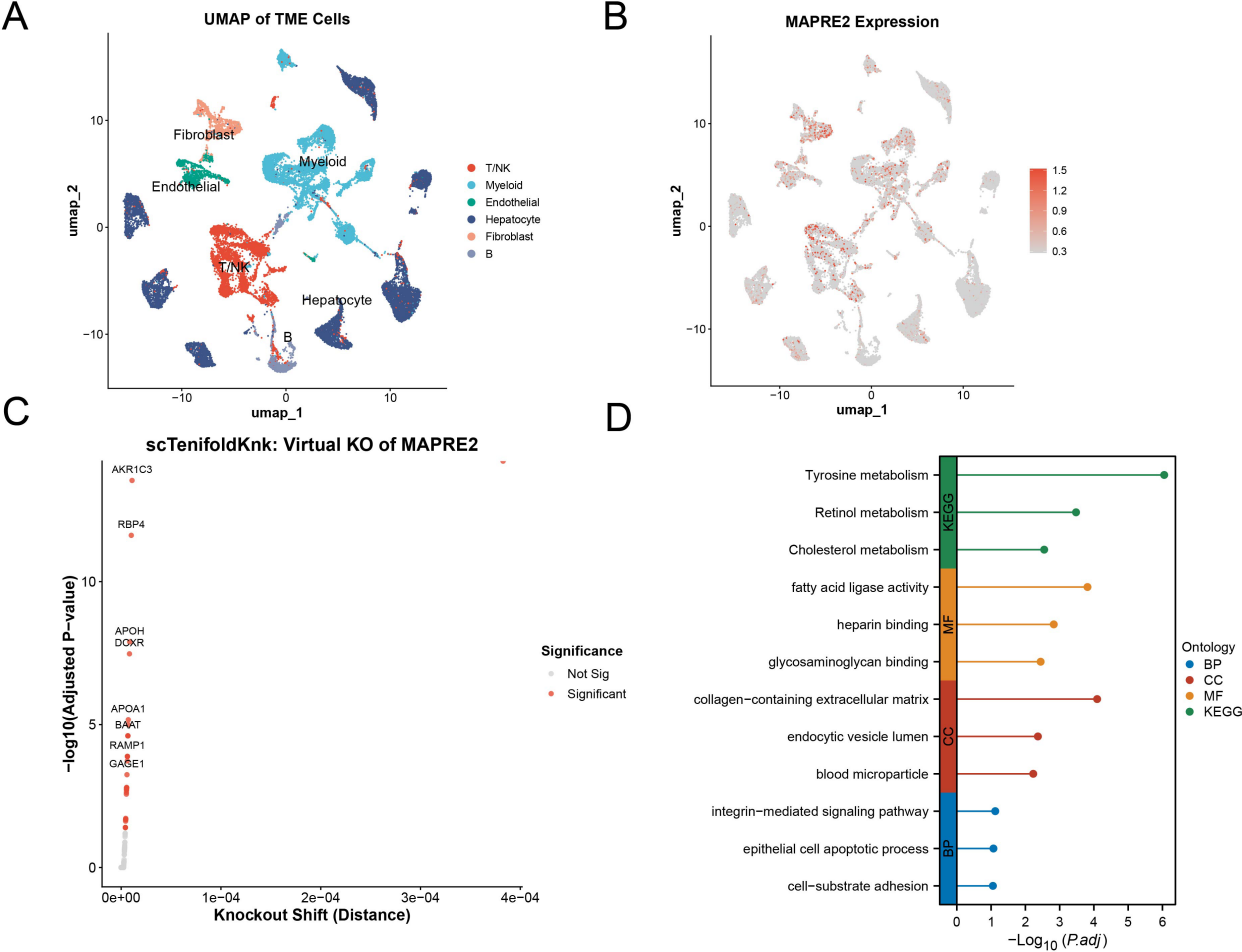
Single-cell transcriptomic analysis and virtual knockout implicate MAPRE2 in multicellular hepatocellular carcinoma programs. **(A)** UMAP of tumor microenvironment cells from GSE149614 annotated as T/NK, myeloid, endothelial, hepatocyte, fibroblast, and B cells. **(B)** Feature plot showing MAPRE2 expression across cell clusters. **(C)** scTenifoldKnk virtual knockout analysis of MAPRE2. Each point represents a gene, positioned by knockout shift distance and adjusted P value; selected significant genes are labeled. **(D)** Functional enrichment analysis of genes significantly perturbed by virtual MAPRE2 knockout. BP, biological process; CC, cellular component; MF, molecular function; KEGG, Kyoto Encyclopedia of Genes and Genomes.

Bulk transcriptomic analyses cannot determine whether MAPRE2 is restricted to malignant hepatocytes or distributed more broadly across the tumor ecosystem. We therefore analyzed the single-cell RNA sequencing dataset GSE149614 restricted to primary tumor-derived cells. Unsupervised clustering resolved major tumor microenvironment compartments including T/NK cells, myeloid cells, endothelial cells, fibroblasts, hepatocytes, and B cells (**Figure 5A**).

Feature plotting showed that MAPRE2 expression was distributed across several compartments rather than being confined to a single cluster, with visible expression in myeloid and stromal regions in addition to hepatocyte-containing areas (**Figure 5B**). To probe possible network-level consequences of MAPRE2 loss, we performed virtual knockout analysis with scTenifoldKnk. MAPRE2 perturbation significantly shifted a subset of genes, among which AKR1C3, RBP4, APOH, DCXR, APOA1, BAAT, RAMP1, and GAGE1 were among the labeled responses with the strongest significance (**Figure 5C**). Enrichment analysis of perturbation-responsive genes highlighted tyrosine metabolism, retinol metabolism, cholesterol metabolism, fatty acid ligase activity, glycosaminoglycan and heparin binding, collagen-containing extracellular matrix, endocytic vesicle lumen, blood microparticle, cell-substrate adhesion, epithelial cell apoptotic process, and integrin-mediated signaling (**Figure 5D**). These findings support a multicompartment role for MAPRE2 in HCC-associated metabolic and extracellular interaction programs.

### 3.6 MAPRE2 knockdown suppresses proliferation of HCC cells

**Figure 6:**
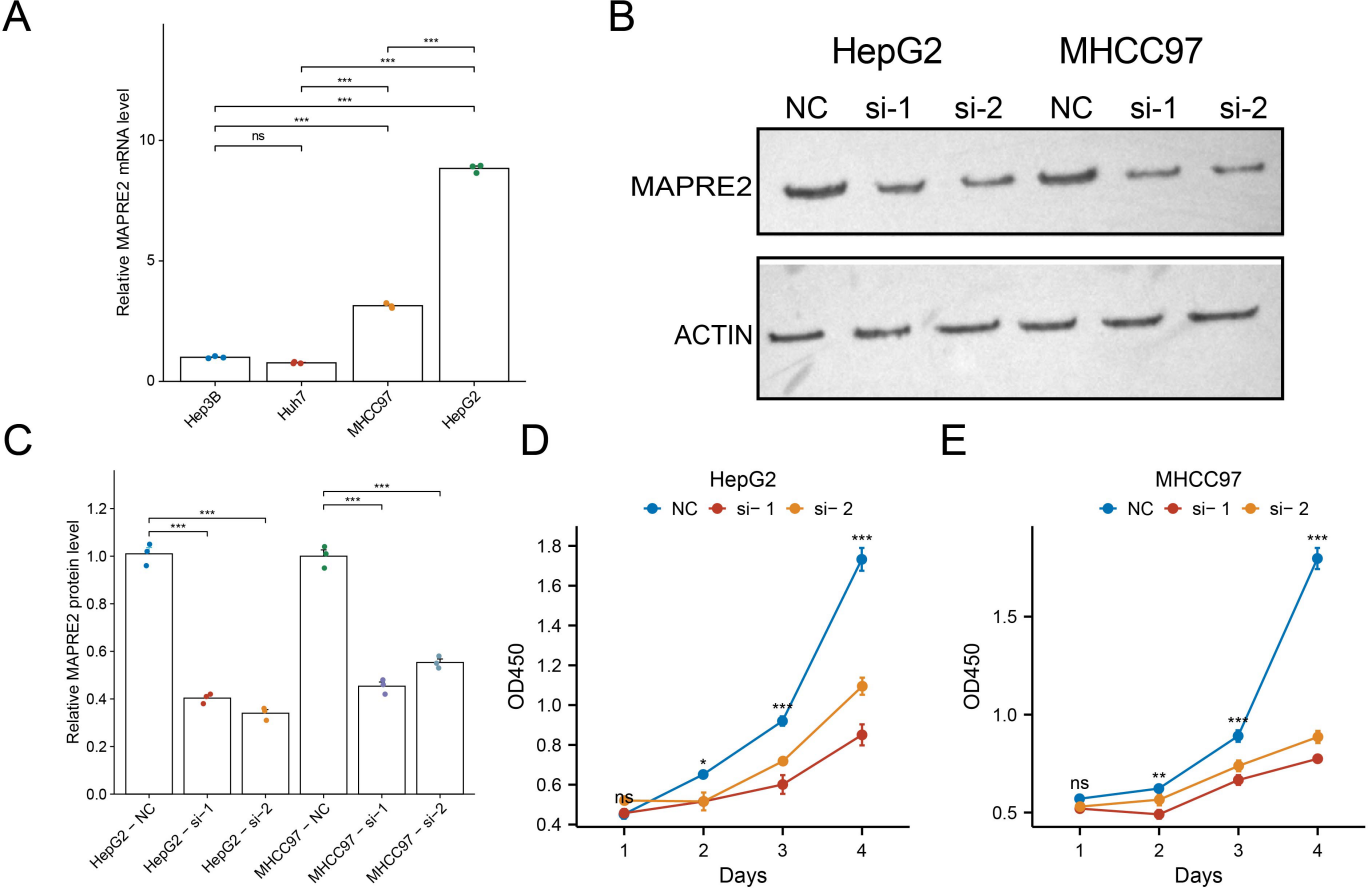
MAPRE2 knockdown suppresses proliferation of hepatocellular carcinoma cells. **(A)** Basal MAPRE2 mRNA expression in four hepatocellular carcinoma cell lines measured by RT-qPCR. **(B)** Western blot validation of MAPRE2 knockdown in HepG2 and MHCC97 cells transfected with NC, negative control, si-1, or si-2. The full, uncropped scans of the original gels are provided in **Supplementary Figure 2**. **(C)** Densitometric quantification of MAPRE2 protein normalized to ACTIN. **(D, E)** Viability trajectories assessed by CCK-8 assay in MAPRE2-depleted HepG2 **(D)** and MHCC97 **(E)** populations. Values represent the mean ± SEM across three biological replicates.

We next sought direct experimental support for a functional role of MAPRE2 in HCC cells. Basal MAPRE2 mRNA expression was measured in four hepatocellular carcinoma cell lines. HepG2 and MHCC97 cells expressed substantially higher MAPRE2 than Hep3B and Huh7 cells and were therefore selected for loss-of-function experiments (**Figure 6A**).

Two independent small interfering RNAs efficiently reduced MAPRE2 protein in both HepG2 and MHCC97 cells, as shown by western blotting and densitometric quantification (**Figures 6B, C**; original uncropped blots are shown in **Supplementary Figure 2**). CCK-8 assays demonstrated that MAPRE2 silencing significantly impaired cell growth in both cell lines. In HepG2 cells, the effect became evident by day 2 and was marked at days 3 and 4, whereas in MHCC97 cells growth inhibition was apparent from day 2 onward and became more pronounced thereafter (**Figures 6D, E**). These data support a role for MAPRE2 in sustaining HCC cell proliferation.

### 3.7 MAPRE2 knockdown impairs invasion and migratory wound closure

**Figure 7:**
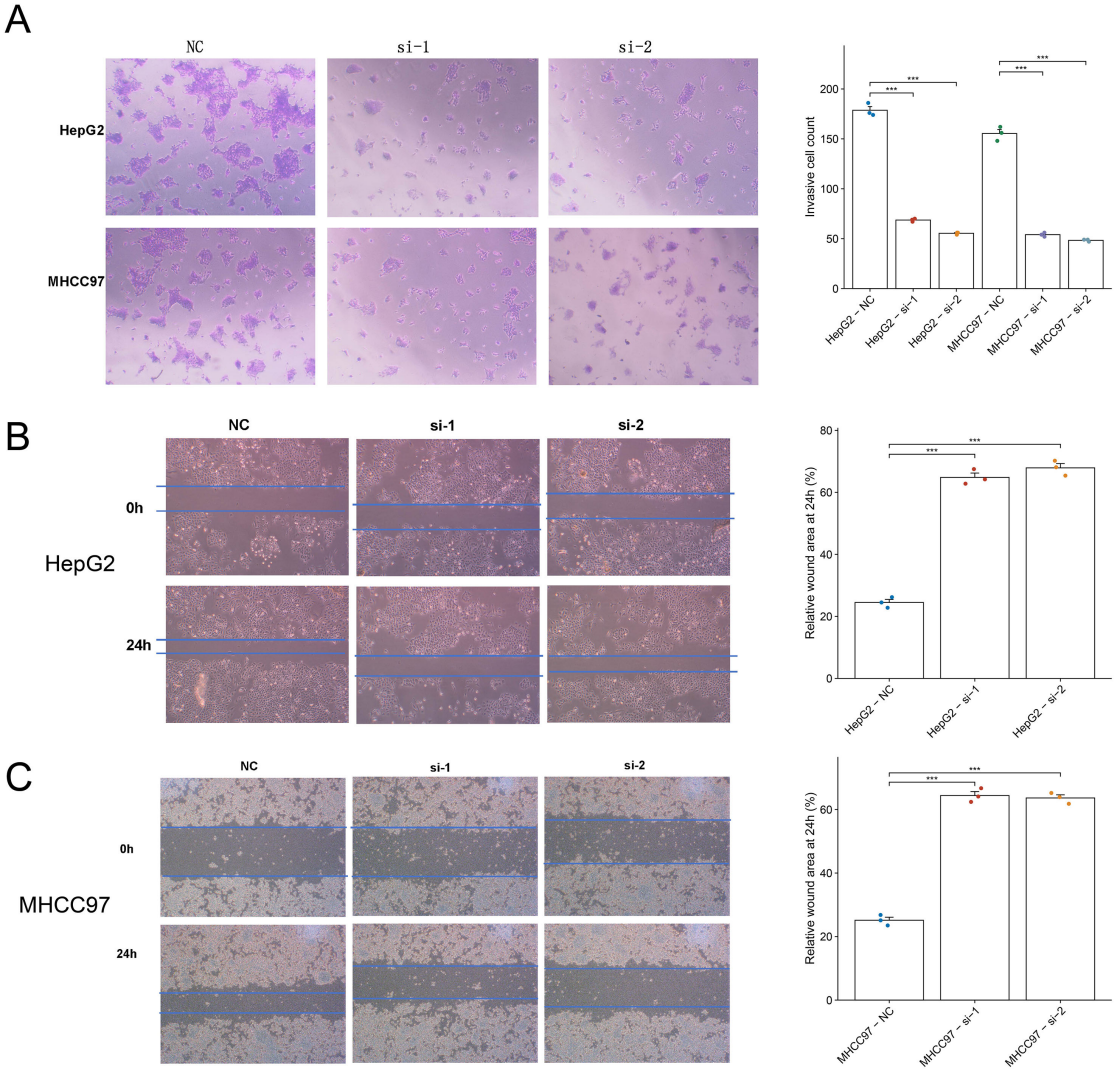
MAPRE2 knockdown reduces invasion and migration of hepatocellular carcinoma cells. **(A)** Representative Transwell invasion images and quantification for HepG2 and MHCC97 cells after MAPRE2 silencing. **(B)** Representative wound-healing images and quantification of residual wound area at 24 h in HepG2 cells. **(C)** Representative wound-healing images and quantification of residual wound area at 24 h in MHCC97 cells. Data are mean ± SEM from three independent experiments. ***P < 0.001.

To determine whether MAPRE2 also contributes to invasive and migratory behavior, we performed Transwell invasion assays and wound-healing assays after siRNA-mediated silencing. In both HepG2 and MHCC97 cells, MAPRE2 knockdown markedly reduced the number of invading cells compared with negative control cells (**Figure 7A**).

Consistent with this result, wound-healing assays showed that residual wound area at 24 h was significantly larger in the MAPRE2-silenced groups than in the control group in both HepG2 and MHCC97 cells (**Figures 7B, C**). Thus, MAPRE2 depletion delayed wound closure and diminished invasive capacity, in agreement with the transcriptomic evidence linking MAPRE2 to extracellular matrix remodeling and epithelial-mesenchymal transition-related programs.

### 3.8 Exploratory pharmacogenomic analysis links MAPRE2 expression to broad drug-sensitivity patterns

**Figure 8:**
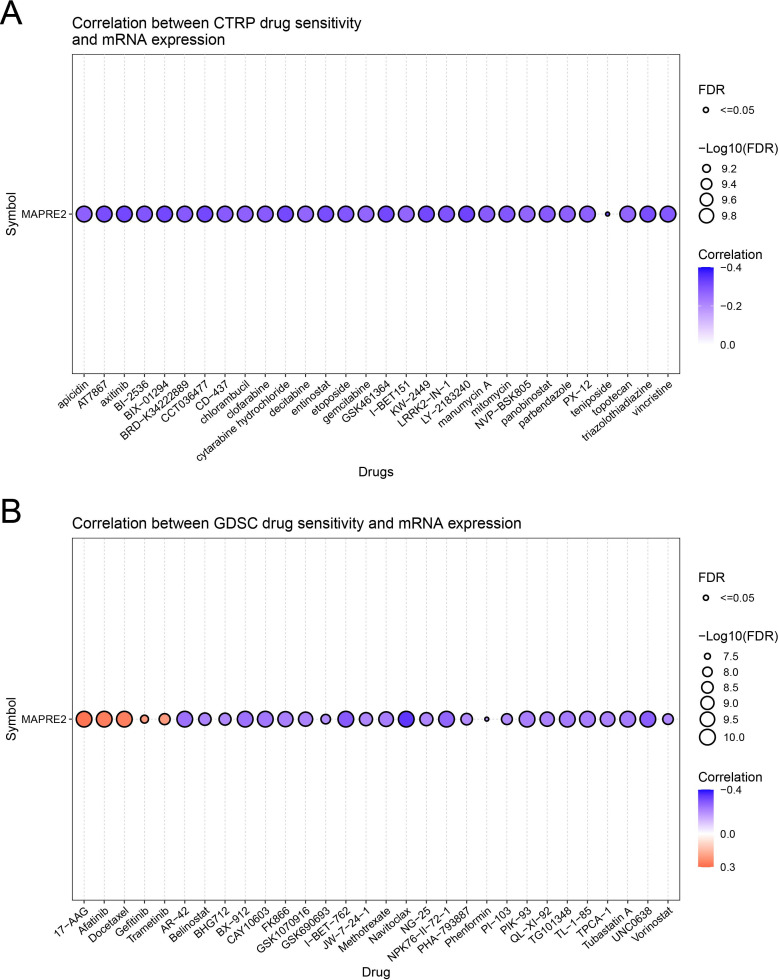
Exploratory pharmacogenomic associations of MAPRE2 expression with drug sensitivity. **(A)** Correlation between MAPRE2 mRNA expression and CTRP drug-sensitivity readouts. **(B)** Correlation between MAPRE2 mRNA expression and GDSC drug-sensitivity readouts. Bubble size indicates -log10(FDR), and bubble color indicates the correlation coefficient. These analyses are exploratory and hypothesis-generating.

To explore whether MAPRE2 expression might be associated with pharmacologic response patterns, we used GSCA to interrogate CTRP and GDSC drug-sensitivity datasets. In CTRP, MAPRE2 expression showed predominantly negative correlations across a broad range of compounds (**Figure 8A**). GDSC showed a mixed but still largely negative pattern, although a limited subset of agents, including 17-AAG, afatinib, docetaxel, gefitinib, and trametinib, showed positive correlations (**Figure 8B**). These associations should be interpreted as hypothesis-generating rather than definitive, but they suggest that MAPRE2 expression may help mark distinct pharmacologic response states in HCC-related models.

## 4 Discussion

This study integrated multi-omics Mendelian randomization, variant-level visualization, somatic and epigenetic analyses, bulk and single-cell transcriptomics, innate immune characterization, and *in vitro* validation to evaluate MAPRE2 in hepatocellular carcinoma. Several findings converged. First, MAPRE2 was one of only two genes with concordant risk associations in both eQTL-based and pQTL-based MR. Second, single-variant plots showed that this signal was not being driven by a single idiosyncratic instrument, but instead reflected a directionally consistent pattern across multiple regulatory variants. Third, MAPRE2 was reproducibly overexpressed in tumors, associated with worse survival in exploratory survival analysis, and supported by borderline higher CPTAC protein abundance. Fourth, recurrent somatic SNV/CNV events did not appear to explain its dysregulation, whereas methylation-associated analyses suggested a more plausible regulatory layer. Finally, knockdown experiments demonstrated that MAPRE2 supports proliferation, invasion, and migration of HCC cells. Together, these findings place MAPRE2 at the intersection of malignant progression and innate immune dysregulation in HCC.

A major strength of the study is that candidate genes were prioritized using both transcript-level and protein-level genetic instruments. Requiring directional agreement across eQTL and pQTL analyses reduced the candidate list and increased confidence that the signal was not specific to a single platform or tissue readout. By additionally visualizing the retained instruments for MAPRE2, we made the variant-level support more explicit than in a gene-level MR summary alone. This is important because susceptibility-gene prioritization is stronger when the reader can see that multiple inherited regulatory variants point in the same direction. At the same time, it should be acknowledged that we did not perform formal colocalization, fine-mapping, or experimental dissection of the individual regulatory variants. Therefore, the present study should be interpreted as strong prioritization rather than definitive proof of the exact causal variant. In addition, the use of blood-derived eQTLs and plasma pQTLs may limit tissue-specific interpretation. These instruments may reflect systemic genetically regulated transcript or protein abundance rather than regulatory activity within hepatocytes, tumor cells, liver-resident macrophages, or the HCC microenvironment. Therefore, the MR results should be interpreted as genetic prioritization evidence for MAPRE2 rather than definitive proof of a liver-specific causal regulatory mechanism.

An equally important result is what we did not find. MAPRE2 does not appear to behave like a classic recurrent tumor hotspot gene in LIHC. High-level amplification was rare in cBioPortal, GSCA showed mainly low-level heterozygous copy-number events, and neither CNV-expression nor SNV-expression analyses were significant. This negative result is informative. It suggests that MAPRE2 upregulation in HCC is unlikely to be driven primarily by frequent somatic genomic alteration. Instead, the inverse relationship between site-specific methylation and MAPRE2 expression, together with methylation-associated survival differences, supports a model of regulatory and epigenetic dysregulation. The tumor-normal methylation effect size was modest, so we do not interpret this as a fully resolved mechanism, but the overall pattern is more compatible with regulatory imbalance than with recurrent mutational activation.

The bulk transcriptomic, immune, and single-cell analyses place this genetically prioritized signal in an innate-immune context. MAPRE2-high tumors were enriched for extracellular matrix remodeling, inflammatory signaling, and epithelial-mesenchymal transition-related programs. They also showed immune checkpoint upregulation, macrophage-related infiltration, and lower immunophenoscores, suggesting an inflamed but potentially ineffective immune state rather than a productive antitumor response. Single-cell analysis further showed that MAPRE2 was distributed across multiple tumor microenvironment compartments rather than being limited to one cluster, and virtual knockout implicated metabolic, adhesion-related, and extracellular programs. These results do not by themselves define a direct mechanism, but they help localize MAPRE2 within the innate immune and stromal context in which aggressive HCC develops.

MAPRE2 should also be interpreted in a broader pan-cancer context. Recent TCGA-based pan-cancer biomarker studies have emphasized that survival-associated genes may be shared across malignancies or may show cancer-type-specific prognostic directions, and that differential expression alone is insufficient for clinical biomarker designation without tumor-specific survival and functional validation (25). Consistent with this concept, we do not interpret MAPRE2 as an HCC-exclusive biomarker. Rather, the present data support an HCC-focused role in which genetically prioritized MAPRE2 expression is coupled to macrophage-enriched innate immune dysregulation, extracellular matrix remodeling, and malignant phenotypes. Comparative pan-cancer analyses will be needed to determine whether the MAPRE2-associated innate immune program observed here is liver-specific or reflects a broader oncogenic mechanism.

The potential relevance of MAPRE2 to liver transplantation also deserves consideration. Liver transplantation remains a curative option for selected patients with HCC, but post-transplant recurrence remains clinically important, and molecular, serological, and microbiome-associated biomarkers have been investigated to refine transplant-related risk stratification (26–28). In the present study, MAPRE2-high tumors showed enrichment of epithelial-mesenchymal transition, extracellular matrix remodeling, macrophage/M2-like signatures, and immunosuppressive features. These observations raise the possibility that MAPRE2 could be evaluated in future explant-tissue or pre-transplant biopsy cohorts as a complementary molecular marker for recurrence risk or post-transplant surveillance intensity. However, the current TCGA-LIHC dataset does not include transplant-specific outcomes, immunosuppressive regimens, graft-related variables, or post-transplant recurrence data. Therefore, any role of MAPRE2 in transplant eligibility, transplant-risk stratification, or post-transplant recurrence prediction remains hypothesis-generating and requires dedicated validation.

The functional experiments close the loop between genetic prioritization and tumor phenotype. In HepG2 and MHCC97 cells, MAPRE2 knockdown reduced growth, invasion, and wound closure, consistent with the previously reported role of EB2 in HCC proliferation and metastasis (8). Our exploratory pharmacogenomic analysis further suggested that MAPRE2 expression tracks broad drug-response patterns across CTRP and GDSC, although these findings remain hypothesis-generating and should not yet be interpreted as predictive biomarkers for clinical therapy.

Several limitations should be considered. First, the MR analyses relied on blood-derived eQTLs and plasma pQTLs rather than liver-, hepatocyte-, macrophage-, or tumor-specific QTL datasets. These instruments may capture systemic genetically regulated expression or circulating protein abundance and may not fully reflect regulatory activity within the HCC tissue microenvironment. Therefore, the MR findings should be interpreted as genetic prioritization evidence rather than definitive proof of an HCC-specific causal regulatory mechanism. Second, we did not perform formal colocalization, fine-mapping, allele-specific functional assays, methylation perturbation experiments, plasmid-based rescue experiments, or *in vivo* validation. Although two independent siRNAs reduced MAPRE2 protein and produced concordant suppression of proliferation, invasion, and wound closure, rescue experiments using an siRNA-resistant MAPRE2 construct will be needed to fully exclude off-target effects. Third, macrophage infiltration and M2-like polarization were inferred from bulk immune deconvolution, immune-signature analysis, and single-cell transcriptomic context rather than directly validated in tumor tissue after MAPRE2 perturbation. Orthotopic, syngeneic, or humanized HCC models with MAPRE2 knockdown or knockout, followed by macrophage immunostaining or flow cytometry, will be required to test whether MAPRE2 directly reshapes macrophage infiltration or polarization *in vivo*. Fourth, the current public datasets do not include liver-transplant-specific outcomes, post-transplant recurrence, immunosuppressive regimens, or graft-related variables, limiting conclusions regarding transplantation. Finally, the survival analyses should be externally validated in clinically annotated cohorts before MAPRE2 can be considered an independent prognostic or transplant-risk biomarker. Even with these limitations, the convergence across inherited regulatory data, tumor expression, methylation, protein abundance, innate immune context, and cell-based phenotype supports MAPRE2 as a genetically prioritized HCC-associated candidate linked to macrophage-enriched innate immune dysregulation and aggressive malignant phenotypes.

## Data Availability

Publicly available datasets used in this study can be obtained from TCGA-LIHC through the Genomic Data Commons, from the eQTLGen consortium, from the deCODE plasma proteogenomic resource, from the GWAS Catalog under accession GCST90043858, and from GEO under accession GSE149614. Additional regulatory, somatic, methylation, pharmacogenomic, and proteomic exploration used cBioPortal, GSCA, and UALCAN/CPTAC. Detailed single-variant eQTL and pQTL MR outputs were compiled in a supplementary Excel file accompanying the article.

## Supplementary material

Supplementary Figure 1 cBioPortal OncoPrint of MAPRE2 alterations in TCGA-LIHC. The default display highlights that high-level MAPRE2 amplification is rare in LIHC and supports the conclusion that recurrent somatic alteration is unlikely to be the primary explanation for MAPRE2 dysregulation.

Supplementary Figure 2 Original uncropped full-scan western blot images for **Figure 6B**.
