# Correction: MAPRE2 is associated with macrophage-enriched innate immune dysregulation and malignant phenotypes in hepatocellular carcinoma

**DOI:** 10.3389/fimmu.2026.1904303

**Published:** 2026-06-17

**Authors:** Xiuqin An, Yanfang Gao, Shangyumeng Zhao, Wei He, Xinda Yang, Yujie Wu, Jihao Li, Runxi Yi, Yue Han, Mingxuan Li, Qiusheng Li, Ting Yang

**Affiliations:** 1 Department of Gastroenterology, First Hospital of Shanxi Medical University, Taiyuan, Shanxi, China; 2 Department of Gastroenterology, Shanxi Provincial People’s Hospital, Taiyuan, Shanxi, China; 3 Department of Preventive Medicine, College of Public Health, Hebei Medical University, Shijiazhuang, China; 4 Department of Hepatobiliary Surgery, The Second Hospital of Hebei Medical University, Shijiazhuang, China

**Keywords:** complement, hepatocellular carcinoma, innate immunity, macrophages, MAPRE2, Mendelian randomization, single-cell RNA sequencing, tumor microenvironment

There was a mistake in **Figure 7A** as published. During final figure assembly using Adobe software, the representative Transwell invasion microscopy image for one si-1 condition was inadvertently not replaced with the correct original microscopy image. The multi-panel layout was generated by copying panel templates and replacing the image layer/source file for each experimental condition. Because the image file names for the two si-1 groups were similar, the same representative Transwell microscopy image was inadvertently placed in both the HepG2-si-1 and MHCC97-si-1 positions. Specifically, the image for the HepG2-si-1 group was mistakenly duplicated in the MHCC97-si-1 panel.

**Figure 7:**
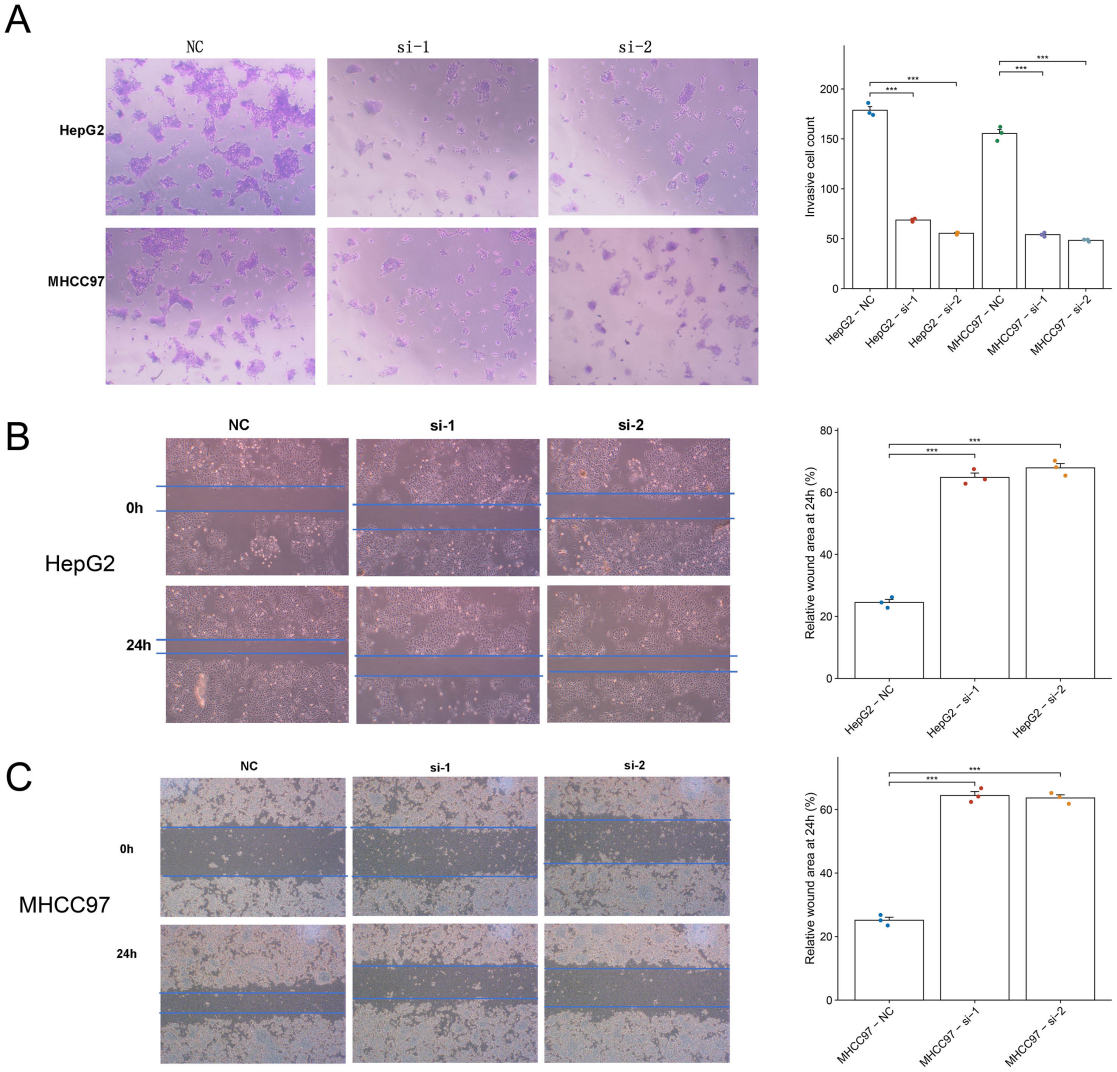
MAPRE2 knockdown reduces invasion and migration of hepatocellular carcinoma cells. **(A)** Representative Transwell invasion images and quantification for HepG2 and MHCC97 cells after MAPRE2 silencing. **(B)** Representative wound-healing images and quantification of residual wound area at 24 h in HepG2 cells. **(C)** Representative wound-healing images and quantification of residual wound area at 24 h in MHCC97 cells. Data are mean ± SEM from three independent experiments. ***P < 0.001.

The corrected **Figure 7** appears below, where the MHCC97-si-1 panel has been updated with the correct original image.

The authors have rechecked the original unprocessed microscopy files, group records, Transwell cell-count data, and statistical analyses. The error is limited to the representative microscopy image displayed in **Figure 7A**. The quantitative cell-count data, statistical values, significance levels, figure legend, results, and conclusions of the article remain unchanged.

The original version of this article has been updated.

