# Supplementary figures and images for: MAPRE2 is associated with macrophage-enriched innate immune dysregulation and malignant phenotypes in hepatocellular carcinoma
Source: Front Immunol. 2026 May 14;17:1849407. doi: 10.3389/fimmu.2026.1849407 (PMC13215804; doi:10.3389/fimmu.2026.1849407)

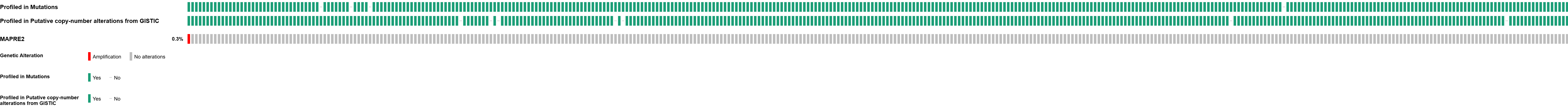

Supplement: Supplementary Figure 1 — cBioPortal OncoPrint of MAPRE2 alterations in TCGA-LIHC. The default display highlights that high-level MAPRE2 amplification is rare in LIHC and supports the conclusion that recurrent somatic alteration is unlikely to be the primary explanation for MAPRE2 dysregulation. [file Image1.png]

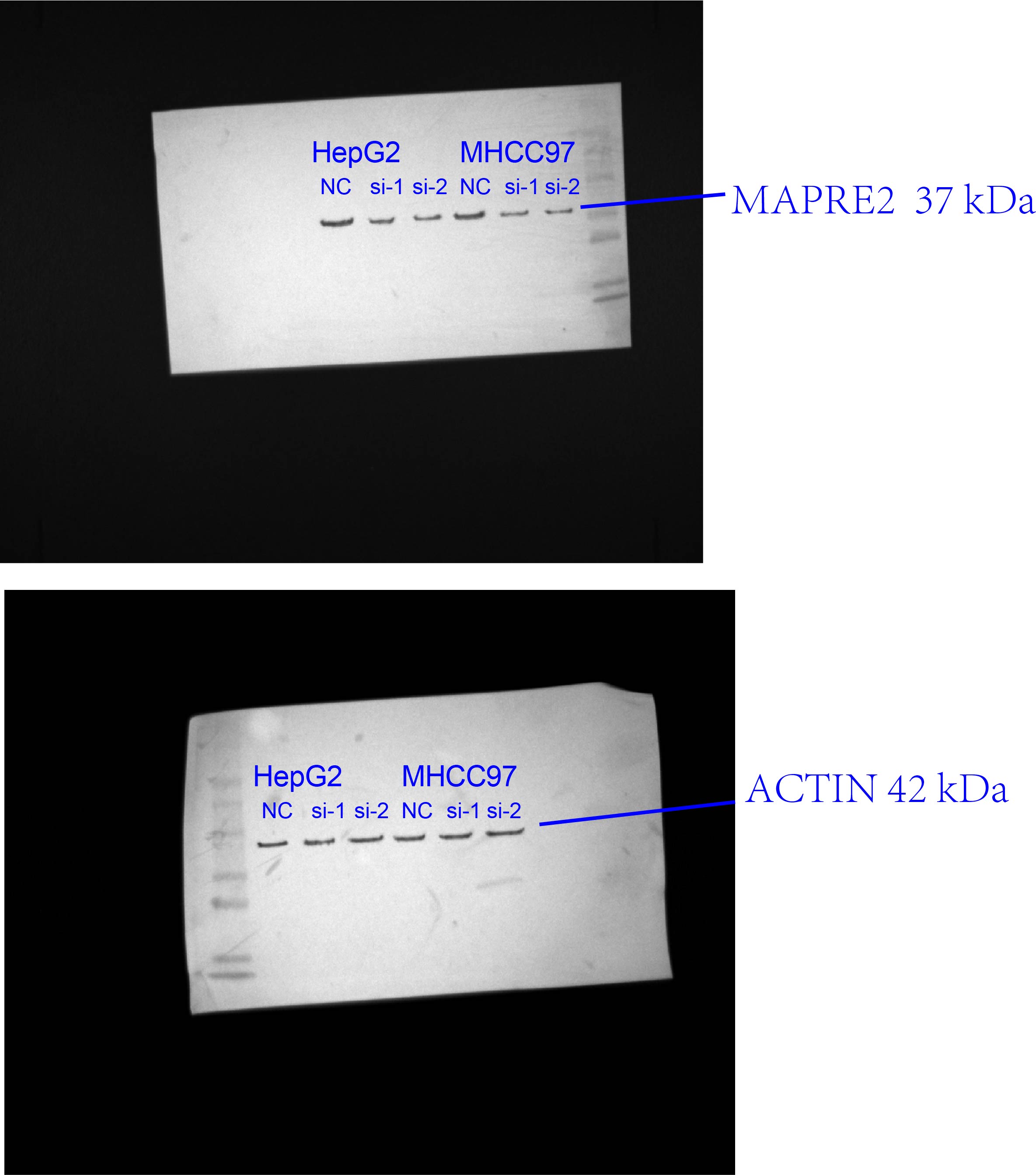

Supplement: Supplementary Figure 2 — Original uncropped full-scan western blot images for Figure 6B. [file Image2.jpeg]
